# Characterization and biological evaluation of a novel silver nanoparticle-loaded collagen-chitosan dressing

**DOI:** 10.1093/rb/rbaa008

**Published:** 2020-03-30

**Authors:** Rongfu Li, Zhaorong Xu, Qiong Jiang, Yunquan Zheng, Zhaohong Chen, Xiaodong Chen

**Affiliations:** r1 Fujian Burn Institute, Fujian Medical University Union Hospital, Fuzhou, Fujian 350001, China; r2 Fujian Burn Medical Center, Fujian Medical University Union Hospital, Fuzhou, Fujian 350001, China; r3 Fujian Provincial Key Laboratory of Burn and Trauma, Fujian Medical University Union Hospital, Fuzhou, Fujian 350001, China; r4 Department of Critical Care Medicine, Quanzhou First Hospital Affiliated Fujian Medical University, Quanzhou, Fujian 362000, China; r5 Institute of Pharmaceutical Biotechnology and Engineering, Fuzhou University, Fuzhou, Fujian 350001, China

**Keywords:** wound dressing, collagen, chitosan, silver nanoparticles, wound healing, cytokines

## Abstract

Effective coverage and protection is a priority in wound treatment. Collagen and chitosan have been widely used for wound dressings due to their excellent biological activity and biocompatibility. Silver nanoparticles (AgNPs) have a powerful antibacterial effect. In this study, a macromolecular and small-molecular collagen mixed solution, a macromolecular and small-molecular chitosan mixed solution were prepared, and a silver nanoparticle-loaded collagen-chitosan dressing (AgNP-CCD) has been proposed. First, the effects of a collagen-chitosan mixed solution on the proliferation of human umbilical vein endothelial cells and the secretion of cytokines were evaluated. Then, the characteristics and antibacterial effects of the AgNP-CCD were tested, and the effects on wound healing and the influence of wound cytokine expression were investigated via a deep second-degree burn wound model. The results showed that at the proper proportion and concentration, the collagen-chitosan mixed solution effectively promoted cell proliferation and regulated the levels of growth factors (vascular endothelial growth factor [VEGF], epidermal growth factor [EGF], platelet-derived growth factor [PDGF], transforming growth factor [TGF-β1], basic fibroblastic growth factor [bFGF]) and inflammatory factors (TNF-α, IL-1β, IL-6, IL-8). Moreover, AgNP solutions at lower concentrations exerted limited inhibitory effects on cell proliferation and had no effect on cytokine secretion. The AgNP-CCD demonstrated satisfactory morphological and physical properties as well as efficient antibacterial activities. An *in vivo* evaluation indicated that AgNP-CCD could accelerate the healing process of deep second-degree burn wounds and played an important role in the regulation of growth and inflammatory factors, including VEGF, EGFL-7, TGF-β1, bFGF, TNF-α and IL-1β. This AgNP-CCD exerted excellent biological effects on wound healing promotion and cytokine expression regulation.

## Introduction

Skin is the largest organ of the human body. After a burn injury, the maintenance and regulation of the water-electrolyte balance are damaged. Meanwhile, pathogens usually colonize on the wound surface and can easily invade deep tissues and cause a series of pathophysiological changes. Medical dressings can cover wounds caused by burns, trauma and gangrene, aiming to protect the wound from the external environment and working as a temporary barrier, which maintains moist conditions and promotes wound healing. Effective protection and coverage of the wound is the priority in wound treatment, so the appropriate choice of wound dressing is very important.

Collagen is the main component of the extracellular matrix and plays an important role in the preparation of medical dressings due to its excellent biological activity and biocompatibility [1]. Given its unique molecular structure and functions, collagen has been widely used for artificial blood vessels and regenerative scaffolds [2, 3]. Previous studies have shown that collagen can promote wound healing by increasing the expression of local cytokines such as vascular endothelial growth factor (VEGF), epidermal growth factor (EGF) [4], platelet-derived growth factor (PDGF) and transforming growth factor (TGF-β) [5].

Chitosan is a kind of biomedical material applied as a wound dressing because of its great biocompatibility and biodegradability and excellent haemostatic and antibacterial effects, and it is also widely used in drug release systems and tissue engineering scaffolds [6–8]. Chitosan can significantly promote wound haemostasis by enhancing platelet adhesion and aggregation [9] and promote wound healing by reducing the inflammatory response of the wound, improving vascular endothelial proliferation and increasing the expression of cytokines and growth factors [10]. Additionally, chitosan can enhance chemotaxis and the function of inflammatory cells to accelerate the formation of granulation tissue and the wound healing process [11].

Silver, a powerful antibacterial compound with efficient and broad-spectrum antibacterial activity, has been used for over a hundred years and can resist a variety of gram-positive or gram-negative bacteria, aerobic or anaerobic bacteria, and some antibiotic-resistant strains [12, 13]. In recent years, it has been widely used in various biomedical applications in its nano-size form due to its ability to prevent antibiotic resistance [14]. Currently, a variety of wound dressings containing Ag^+^ or silver nanoparticles (AgNPs) have been developed, and their safety and efficacy have been confirmed [15]. It has also been reported that AgNPs can be applied with chitosan [16] and carbon fibres [17], resulting in better antibacterial effects.

Wound healing is a complex and orderly process that includes an inflammation phase, a granulation tissue phase and a reconstruction phase. Angiogenesis in the granulation tissue phase is a key link in the process of wound healing, which includes the proliferation, migration and differentiation of vascular endothelial cells regulated by a variety of factors, including VEGF, EGF, PDGF, TGF and basic fibroblastic growth factor (bFGF) [18, 19]. It is widely accepted that a moderate inflammatory response is necessary for wound repairment; therefore, appropriate control of wound inflammation would be beneficial for wound healing. An excessive inflammatory response and secretion of inflammatory mediators (TNF-α, IL-1, IL-6, IL-8) would lead to uncontrolled inflammation and further necrosis [20]. Above all, chitosan and collagen have significant roles in the promotion of wound healing, and AgNPs have powerful antibacterial effects. Thus, these three agents share great effects in the prevention of infection and promotion of wound healing and deserve further research on their synergistic effect as a novel wound dressing.

In this study, a collagen solution including macromolecular (the molecular weight was over 80 kDa) and small-molecular (the average molecular weight was 1 kDa) collagen at a specific mass ratio, and a chitosan solution including macromolecular (the molecular weight was over 50 kDa) and small-molecular (the average molecular weight was 2 kDa) chitosan at a specific mass ratio were prepared. Then, a silver nanoparticle-loaded collagen-chitosan dressing (AgNP-CCD) was first prepared with AgNPs and these solutions above. Within this novel dressing, the large molecules of collagen and chitosan together constituted the skeleton of the scaffold, whereas the small molecules of collagen and chitosan dissolved in the skeleton structure. The stability of the skeleton structure was conducive to maintaining high water absorption, and small molecule collagen and chitosan were dissolved into the wound surface to play the role of promoting wound healing. First, the effect of the collagen-chitosan mixed solution on the proliferation of human umbilical vein endothelial cells (HUVECs) and the secretion of growth and inflammatory factors were evaluated. Then, the characteristics and antibacterial effects of AgNP-CCD were tested, and a deep second-degree burn wound rat model was established to investigate the effects of the AgNP-CCD on the wound healing, the influence of wound growth and the inflammatory factor expression.

## Materials and methods

### Materials

A collagen-chitosan mixed solution with a mass ratio of 1:1, a collagen solution (bovine type-I collagen) and a chitosan solution (the deacetylation degree was >90%) were all provided by the Institute of Pharmaceutical Biotechnology and Engineering, Fuzhou University, China. The detail of these solutions was described earlier. These three solutions were serially 2-fold diluted in Dulbecco's Modified Eagle Medium (DMEM), making the final concentrations 2000–0 mg/l, 2500–0 mg/l and 9600–0 mg/l, respectively, for cytological evaluation. The collagen-chitosan dressing (CCD), which was fabricated by freeze-drying the collagen-chitosan mixed solution, was also provided by the Institute of Pharmaceutical Biotechnology and Engineering. The AgNP solutions (particle size <15 nm) were purchased from Shanghai Huzheng Nanotechnology Co. Ltd., China. Dilutions ranging from 15 to 0 mg/l AgNP solution were also made in DMEM for the cytotoxicity tests. Standardized cultures of *Staphylococcus aureus* (ATCC25923), *Escherichia coli* (ATCC25922) and *Pseudomonas aeruginosa* (ATCC27853) were used for the antibacterial studies. Sprague Dawley (SD) rats were used for the *in vivo* investigations of the deep second-degree burn wounds treated with the AgNP-CCD.

### Preparation of AgNP-CCD

The CCDs were prepared in pieces (20 × 20 × 1 mm) and then loaded with AgNPs. In brief, the CCDs were immersed in 1 ml AgNP solution of different concentrations according to the total surface area, to make the final amount of AgNPs loaded 0.15, 0.3, 0.6 or 1.2 mg/cm^2^. After complete immersion and absorption, the AgNP-CCD was obtained. The AgNP-CCDs were sterilized with ethylene oxide and then were ready for the experiments. In this case, AgNPs were loaded simply by soaking without any chemical reactions.

### Cell culture and growth curves

HUVECs were purchased from ScienCell Research Laboratories (USA) and were cultured in DMEM supplemented with 10% foetal calf serum and incubated at 37°C in a humidified incubator with 5% CO_2_ with medium exchanges every 3 days. HUVECs were passaged when they approached 75–80% confluence, and cells between the third and fourth passages were used for further experiments. The cells were digested with 0.25% trypsin (Gibco, USA) and then seeded in 96-well plates (100 μl/well) at a density of 10^5^ cells/ml. The supernatants were removed after incubation for 24 h, and then 100 μl of various concentrations of the prepared solutions were added. Cells cultured in DMEM were used as a control. At serial time intervals (0, 1, 2, 3, 4, 5, 6 and 7 days) after incubation, samples were taken for the Methyl Thiazolyl Tetrazolium assay following the manufacturer’s instructions. The optical densities were measured at 570 and 650 nm using a microplate reader (Molecular Devices MAX 190, USA). Cell growth curves were plotted with time on the horizontal axis and optical density on the vertical axis.

### Determination of cytokine levels

HUVECs at a density of 1.5 × 10^4^ cells/ml were seeded in 24-well plates (500 μl/well) and incubated for 24 h. After the supernatant was removed, 500 μl of certain concentrations of the prepared solutions were added for another incubation of 48 h. The supernatants were then collected for the assay. The concentrations of VEGF, EGF, PDGF, TGF-β1, bFGF, TNF-α, IL-1β, IL-6 and IL-8 were determined by ELISA kits (Shanghai Westing Bio-Tech Co. Ltd., China). The results are reported as the mean of parallel experiments.

For wound tissues, each collected tissue was homogenized in Phosphate Buffer Saline followed by centrifugation at 10 000 RPM for 10 min, and then the supernatants were removed for the assay. The total protein concentration of the samples was determined with BCA kits. The concentrations of VEGF, EGFL-7, TGF-β1, bFGF, TNF-α and IL-1β were determined by ELISA kits. The content of each cytokine in the wound tissue was equal to the concentration measured by ELISA divided by the total protein concentration.

### Antibacterial testing

To evaluate the antibacterial effect of AgNP-CCD, bacterial strains were grown in MH broth overnight, and a suspension of 1.5 × 10^6^ CFU/ml using McFarland standards was prepared. A 3 ml aliquot of each bacterial suspension was then added to each sample (10 × 10 mm), which was placed in a sterile tube while ensuring that the sample was fully saturated. After incubation for 24 h, the suspension was diluted by serial 10-fold dilutions, and a 1 ml aliquot of each dilution was inoculated on an MH agar plate. Colony counts were performed after overnight incubation, thus, obtaining the exact bacterial density of each sample. The antibacterial rate was determined according to the following equation: (A−B)/A × 100% (A: bacterial density after 24 h of incubation and B: inoculum bacterial density). This experiment was repeated in triplicate, and the results are recorded as the mean of the experiments.

### Characterization

#### Silver ion release

An *in vitro* silver ion release study was performed in which 1 × 1 cm of the AgNP-CCD was immersed in distilled water (10 ml) for 7 days. At different time intervals (1, 3 and 7 days) after immersion, each solution was collected to determine the Ag^+^ concentration with inductively coupled plasma mass spectrometry (ICP-MS, Agilent, USA). The results were reported as the mean of the experiment that was conducted in triplicate.

#### Micromorphology

The distribution of AgNPs on the surface of the AgNP-CCD was observed using a Hitachi SU8010 scanning electron microscope (SEM). The CCD was used as a control. Samples were fixed on a stage and coated with a thin layer of gold under vacuum for scanning.

#### Water absorption

Each sample (1 × 1 cm) was immersed in distilled water and weighed every 2 h until the weight no longer increased; then, the weight was defined as W_W_. The weight of each dried sample was recorded as W_S_. The rate of water absorption was determined according to the following equation: (W_W_ − W_S_)/W_S_ × 100%.

### *In vivo* study

Animal experiments were performed in the Laboratory Animal Center, Fujian Medical University, Fuzhou, China, with the animal protocols approved. Rats had free access to food and water and were maintained in a specific pathogen-free environment under 12 h light-dark cycles at 22–24°C. For each rat, after anaesthetization, barbering and sterilization, four scald burn wounds were induced on the back. The skin area was exposed to an aluminium bar 20 mm in diameter for 8 s, which had been preheated in boiling water for 20 min. This deep second-degree burn wound was confirmed pathologically. Fluid resuscitation, consisting of an intraperitoneal injection of 5 ml of lactated Ringer’s solution, was administered immediately following the burn. The rats were randomly assigned to several treatment groups. Dressings were placed on the wounds; saline gauze for group A, CCD for group B and AgNP-CCD for group C. The rats were bound properly and fed separately, with the dressings replaced every other day.

General observations were conducted during the whole process, including mortality, vital signs, mental state, diet and excretion. At different time intervals post-injury, two rats from each group were selected randomly, and the wound healing rate was calculated with transparent grid paper. Wound tissues were collected and divided into two parts (one for determination of cytokine levels and the other for tissue sectioning and Hematoxylin Eosin (HE) staining).

### Statistical analysis

SPSS v. 18.0 software was used for statistical analysis, and the data were recorded as the mean ± SD. One-way ANOVA was used for comparison between groups, and *P *<* *0.05 indicated a significant difference.

## Results

### Characterization

**Figure 1:**
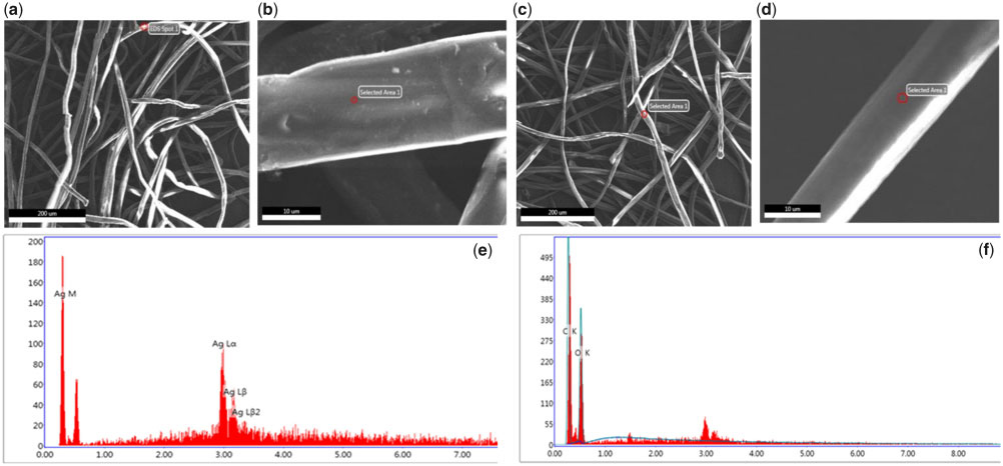
SEM and EDS images of AgNP-CCD and CCD. (**a** and **b**) Compact pore structure with AgNP adhesion. (**c** and **d**) Compact pore structure in the absence of nanoparticles. (**e**) EDS analysis indicating the presence of silver. (f) EDS analysis (silver was not found)

SEM images of the AgNP-CCD are shown in Fig. 1. A compact pore structure was observed in the study area, and AgNPs were observed to be attached to the surface of the fibre. Energy Dispersive Spectrometer (EDS) analysis of random regions of the samples indicated the presence of silver. However, no particles were found in the control group, and the absence of silver was indicated by EDS analysis.

**Figure 2:**
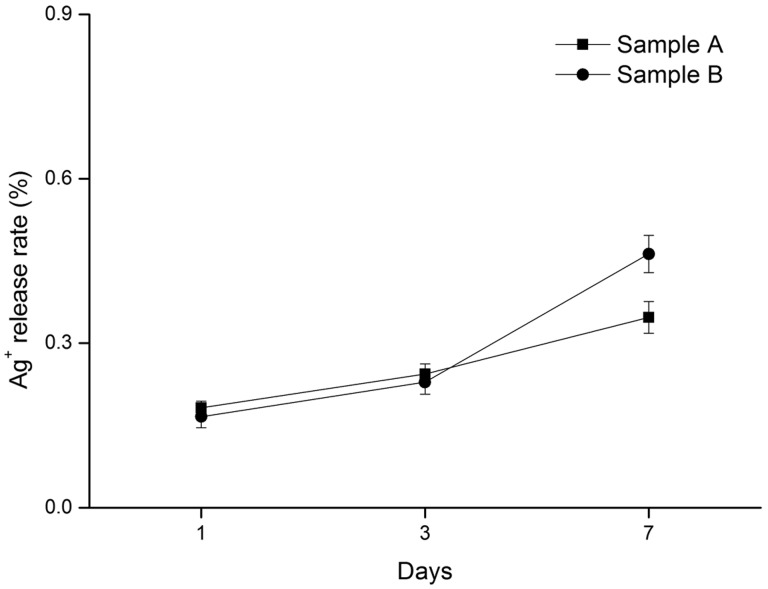
Silver ion release tests (sample A: 0.3 mg/cm^2^, sample B: 0.6 mg/cm^2^)

Silver ion release tests were conducted with samples containing different concentrations of silver ions; sample A (0.3 mg/cm^2^) and sample B (0.6 mg/cm^2^). The results are shown in Fig. 2, which indicates the increasing release rate of silver ions over time.

The water absorption rate of AgNP-CCD was 1471 ± 142%, which showed the advantage of high water absorption.

### Cell growth curves of HUVECs in different culture conditions

**Figure 3:**
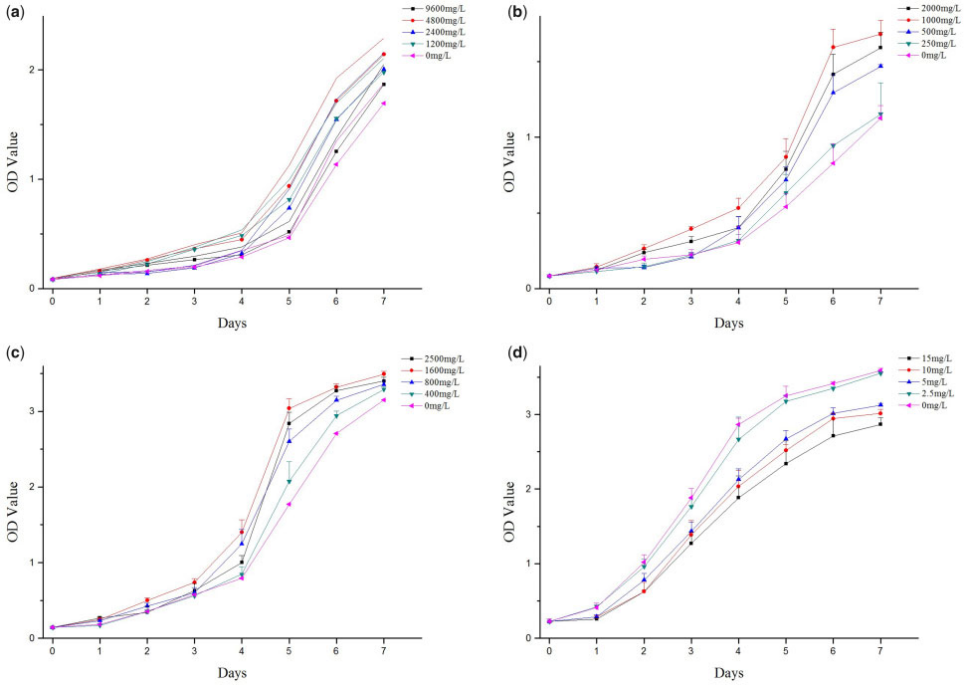
Cell growth curves of HUVECs under different culture conditions. (**a**) Collagen solution; the optimal concentration was 4800 mg/l. (**b**) Chitosan solution; the optimal concentration was 1000 mg/l. (**c**) Collagen-chitosan mixed solution; the optimal concentration was 1600 mg/l. (**d**) AgNP solution; the concentration of limited inhibitory effect was 2.5 mg/l

HUVECs were cultured under different conditions, including a collagen-chitosan mixed solution, a collagen solution, a chitosan solution and an AgNP solution, and the growth curves obtained are presented in Fig. 3. The results (Fig. 3a–c) showed that cell proliferation was significantly promoted by various concentrations of the prepared solutions, which were compared with the control (0 mg/l). The optimal concentrations for cell growth promotion were 4800 mg/l collagen solution, 1000 mg/l chitosan solution and 1600 mg/l collagen-chitosan mixed solution. In contrast to the cell promotion effect, the inhibitory effects of the AgNP solution were observed, which were stronger with increasing concentration (Fig. 3d). The inhibitory effects were not significant compared with the control when the concentration of the AgNP solution was 2.5 mg/l or less.

### HUVECs cytokine levels in different culture conditions

**Figure 4:**
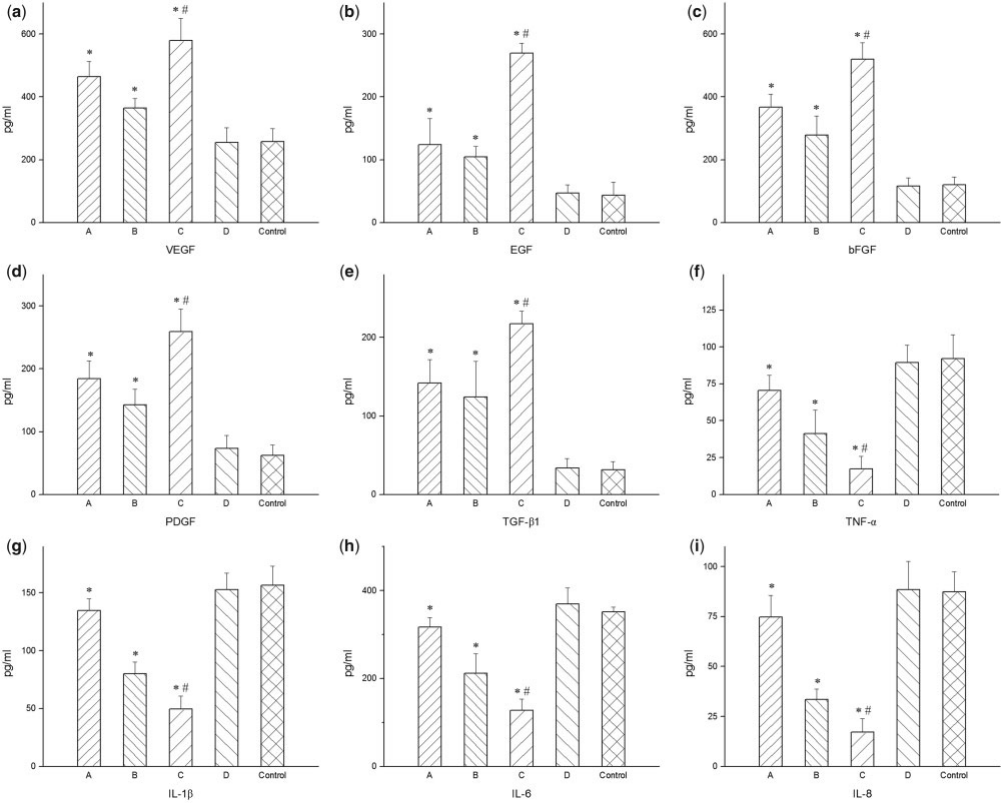
Cytokine levels in HUVECs in different culture conditions. (i) collagen solution (4800 mg/l), (ii) chitosan solution (1000 mg/l), (iii) collagen-chitosan mixed solution (1600 mg/l), (iv) AgNP solution (2.5 mg/l). Growth factors (**a**) VEGF, (**b**) EGF, (**c**) bFGF, (**d**) PDGF and (**e**) TGF-β1 were promoted by the collagen, chitosan and collagen-chitosan mixed solutions compared with the control. Inflammatory factors (**f**) TNF-α, (**g**) IL-1β, (**h**) IL-6, (**i**) IL-8 were inhibited by the collagen, chitosan and collagen-chitosan mixed solutions compared with the control. (**a–i**) The AgNP solution had no effect on the level of growth or inflammatory factors. *Compared with the control group, *P* < 0.05. ^#^Compared with group A or B, *P* < 0.05

The optimal concentration for cell growth of each solution was selected according to the previous results, namely, 4800 mg/l collagen solution, 1000 mg/l chitosan solution, 1600 mg/l collagen-chitosan mixed solution and 2.5 mg/l AgNP solution, for the determination of cytokine levels. As shown in Fig. 4, after incubation for 48 h in the appropriate concentrations of the prepared solutions, the levels of cytokines changed.

The secretion of growth factors (VEGF, EGF, PDGF, TGF-β1, bFGF) was promoted by the collagen solution, chitosan solution and collagen-chitosan mixed solution compared with the control, and the difference was significant (Fig. 4a–e). Furthermore, the collagen-chitosan mixed solution had the strongest effect, which was significantly different from that of the both collagen and chitosan solutions alone. The results also indicated that the AgNP solution had no effect on the levels of growth factors.

The secretion of inflammatory factors (TNF-α, IL-1β, IL-6, IL-8) was inhibited by the collagen solution, chitosan solution and collagen-chitosan mixed solution compared with the control, and the difference was significant (Fig. 4d–i). The inhibitory effect of the collagen-chitosan mixed solution was much stronger than that of collagen solution or chitosan solution. Similar to the results above, the AgNP solution had no effect on the levels of inflammatory factors.

### Antibacterial testing of the dressings

**Figure 5:**
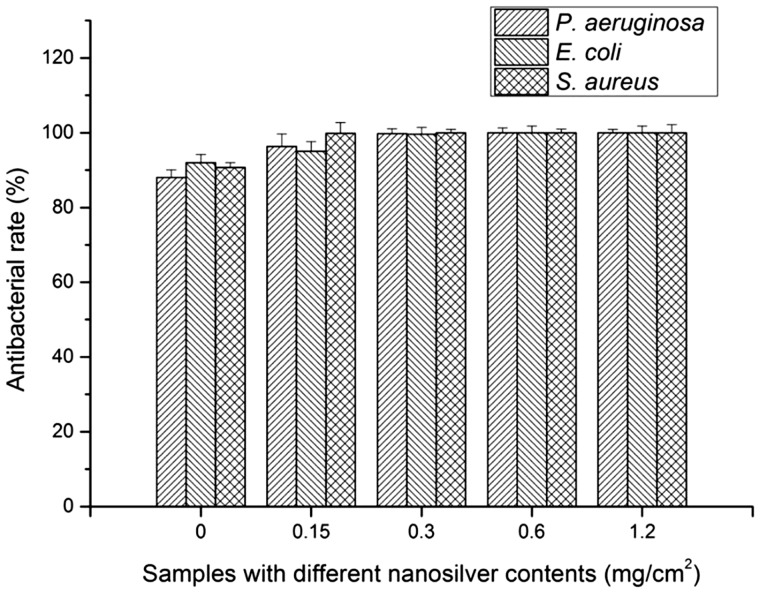
Antimicrobial rates against *S. aureus*, *E. coli* and *P. aeruginosa* of the CCD and AgNP-CCD containing different AgNPs contents

The AgNP-CCD and the CCD were selected to evaluate their antibacterial effects. The results (Fig. 5) showed that the antibacterial rate of the CCD against *S. aureus*, *E. coli* and *P. aeruginosa* was close to or >90% and began to increase with increasing AgNP content, reaching ∼100% when the content was >0.3 mg/cm^2^.

### Evaluation *in vivo*

#### Both CCD and AgNP-CCD accelerate the healing process of the deep second-degree burn wounds

According to the results of silver ion release and antibacterial tests of the dressings, AgNP-CCD (0.6 mg/cm^2^) was selected as group C for the *in vivo* study. Daily observations showed that this dressing was not fatal, and the rats were sensitive and in a good mental state with stable vital signs, normal feeding and normal excretion.

**Figure 6:**
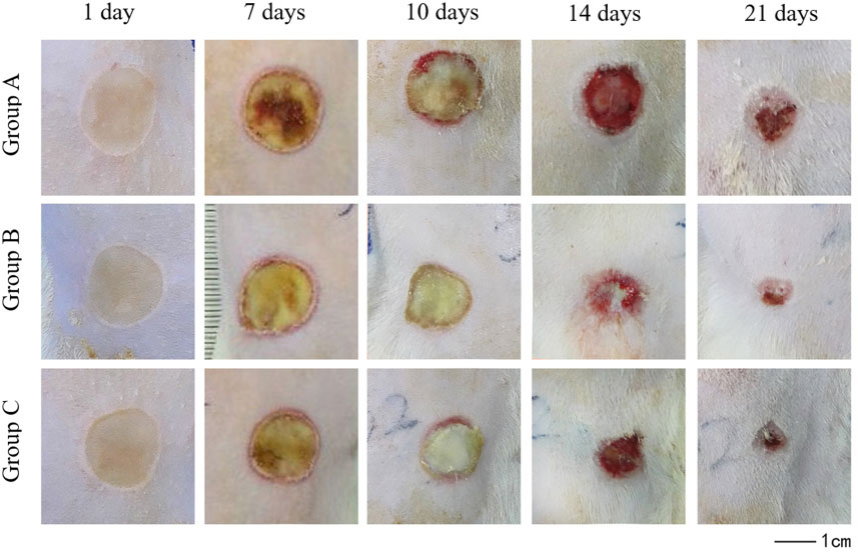
Photographic images showing the wound healing process taken from the same distance on different days.

Images of the wound healing process are shown in Fig. 6. As shown in Table 1, there was no significant difference in the wound healing rate between all three groups 7 days post-scalding, but the rates of healing of groups B and C were significantly higher than those of group A on days 10 and 14. On day 21, the healing rates of groups B and C were close to or >90%, while that of group A was only 60%.

**Table 1:** Wound healing rate (%, & $\bar{x}$ ± s, *n* = 8)

	Time			
Groups	7 days	10 days	14 days	21 days
A (saline gauze)	11.9 ± 1.8	18.1 ± 2.5	38.1 ± 4.6	60.4 ± 7.4
B (CCD)	11.9 ± 1.9	28.7 ± 2.6^a^	58.9 ± 6.8^a^	90.4 ± 7.6^a^
C (AgNP-CCD)	12.3 ± 1.6	29.2 ± 4.1^a^	58.1 ± 6.3^a^	89.8 ± 5.1^a^

^a^ Compared with group A, *P *<* *0.05.

**Figure 7:**
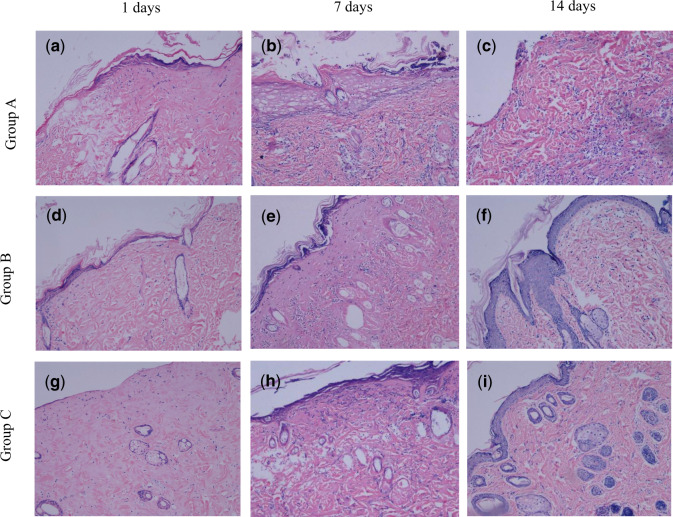
HE Staining of wound tissue sections (100×). (**a–c**) 1, 7 and 14 days after scalding in group A. (**d–f**) 1, 7 and 14 days after scalding in group B. (**g–i**) 1, 7 and 14 days after scalding in group C

Histological observations (Fig. 7) showed that on the first day after scalding, epidermal and dermal tissue necrosis was observed for each group, and a small number of inflammatory cells were found. On Day 7, inflammatory cell infiltration and granulation tissue formation were found in groups B and C, as well as epithelialization at the wound edge, whereas excessive granulation tissue and inflammatory responses were observed in group A. On Day 14, the results of HE staining indicated epithelialization with clear tissue structure in groups B and C. However, the phenomenon of excessive granulation tissue and inflammatory responses still existed in group A, without obvious epithelialization.

#### Growth and inflammatory factors were both regulated by AgNP-CCD

**Figure 8:**
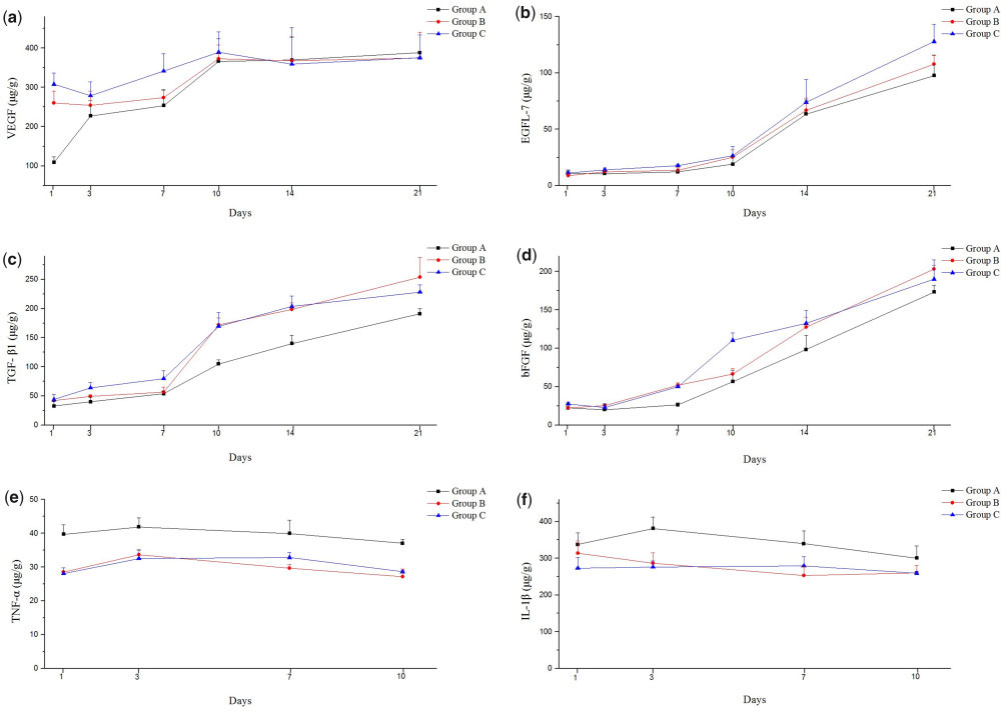
Cytokine levels in wound tissues (group A: saline gauze, group B: CCD, group C: AgNP-CCD). Growth factors (**a**) VEGF, (**b**) EGFL-7, (**c**) TGF-β1, (**d**) bFGF and inflammatory factors, (**e**) TNF-α and (**f**) IL-1β in the wound were both regulated by AgNP-CCD.

The results (Fig. 8a) showed that the VEGF content in both groups B and C was significantly higher than that of group A on the first day after scalding. As time went on, the VEGF content of all three groups increased and reached a maximum value on Day 10, which lasted until Day 21. Other growth factors, including EGFL-7, TGF-β1 and bFGF (Fig. 8b–d), showed no obvious change in all three groups during the first few days after scalding, but the content gradually increased on Day 7 or Day 10 and reached a peak at the end of the experimental period. Moreover, the content of the CCD and AgNP-CCD groups was always higher than that of the control group, but no significant difference was found between the CCD and AgNP-CCD groups.

On the first day after scalding, the levels of the inflammatory factors TNF-α and IL-1β from the CCD and AgNP-CCD groups were much lower than those of the control group, which persisted over the whole healing process (Fig. 8e and f).

## Discussion

Wound healing is affected by many factors, such as inflammation in the early stage and connective and epithelial tissue proliferation in the later stage. Sufficient blood flow and oxygen supply to the wound are related to the number of new blood vessels, which subsequently affects the rate of wound healing. Therefore, angiogenesis plays an important role in the wound healing process [21]. We exposed HUVECs to different concentrations of collagen solution, chitosan solution and collagen-chitosan mixed solution, and the results showed that within the range of certain concentrations, these solutions could significantly stimulate cell proliferation. Many researchers believed that scaffolds prepared from collagen-chitosan had great cytocompatibility, wound healing promotion ability, and improved biostability for tissue engineering [22–24]. In this study, we found that the proper proportion and concentration of collagen-chitosan mixed solution enabled cells to enter the rapid proliferation phase earlier than the collagen or chitosan solutions alone, suggesting that wound dressings prepared from a collagen-chitosan mixed solution in a certain proportion were more conducive to the proliferation of vascular endothelial cells and the formation of new blood vessels during wound healing.

Recently, many studies have focussed on cytokine regulation of the wound healing process for wounds treated with CCDs [25–27]. Therefore, the effects of the solutions and wound dressings on the levels of cytokines were further investigated. TGF-β1 plays an important role in wound healing and scar formation [28], which can promote collagen deposition and tissue fibrosis as well as secretion of extracellular matrix and collagen [29]. In addition, TGF-β1 promotes the healing process by upregulating VEGF. VEGF is a mitotic stimulator and chemotactic factor specific to endothelial cells. As an important regulator of angiogenesis, VEGF can activate vascular endothelial cells, improve local vascular permeability, promote the formation of new blood vessels and play an important role in wound healing and tissue reconstruction [20, 30]. This study found that, after exposure of HUVECs to different culture conditions, the content of TGF-β1 in the collagen-chitosan mixed solution was significantly higher than that in each single solution, and that in each single solution was significantly higher than the control. Similar results have been found from studies of VEGF. EGF can promote the proliferation, differentiation and migration of fibroblasts and vascular endothelial cells in wound tissue [31] as well as promote angiogenesis and epithelial regeneration. Piran *et al.* [32] reported that PDGF is an important polypeptide growth factor produced by a variety of cells that is closely related to cell migration and angiogenesis. BFGF is one of the largest families of growth factors with high biological activities that are currently known, which can regulate the growth and differentiation of endothelial cells and promote the formation of new blood vessels [33]. In this study, it was found that the content of EGF, PDGF and bFGF in the collagen-chitosan mixed solution was significantly higher than those in the single solutions, which was similar to the results obtained for TGF-β1 and VEGF. The above results confirmed that the main components of the dressing could increase growth factors and promote the proliferation of HUVECs and the formation of new blood vessels, which agrees with a literature report [34].

Xie *et al.* [27] showed that the expression of EGF, bFGF and TGF-β increased significantly when rat wounds were treated with CCDs compared with gauze- or chitosan-treated dressings. Similarly, the results of our *in vivo* study showed that the expression of EGF-like domain 7 (EGFL-7) in wound tissue of both the CCD and AgNP-CCD groups were higher than that of the control group at 10 days and later after scalding. EGFL-7 protein is a specific factor excreted by vascular endothelial cells and plays an irreplaceable role in angiogenesis. The lack of the EGFL-7 protein could lead to lumen formation disorder and thus affect the formation of new blood vessels [35]. Moreover, the results of cytokine determination *in vivo* were similar to the results found from the cellular experiments; for instance, the contents of TGF-β1 and VEGF in both the CCD and AgNP-CCD groups were significantly higher after scalding, in addition to bFGF expression, indicating wound healing acceleration effect of the prepared dressings.

Inflammation, as the basic defence of the human body against burn and trauma, is the initial phase of wound healing. Severe trauma, burns, infections or major surgery can activate immune cells, leading to the inflammatory response or even a massive release of inflammatory factors [36]. Mahmoudzadeh *et al.* [25] reported that the release of TNF-α and IL-1β was regulated when macrophages were cultured in 3D collagen-chitosan scaffolds. Sun *et al.* [26] demonstrated that IL-6 secretion from fibroblasts was promoted when cultured in collagen-chitosan scaffolds. TNF-α is a pro-inflammatory factor secreted mainly by mononuclear macrophages. It is critical in the complex inflammatory factor network and plays an important role in inflammatory cascade reactions. Appropriate amounts of TNF-α can significantly stimulate cell proliferation, differentiation, angiogenesis and granulation tissue formation, which is essential for wound repair. IL-1β, which is secreted mainly by activated monocytes and mastocytes with a wide range of physiological effects, is involved not only in various inflammatory reactions but also in cell metabolism and tissue repair [37].

In this study, HUVECs were exposed to different solutions, which were the main components of each dressing, to determine the influence of the solutions on the secretion of inflammatory factors (TNF-α, IL-1β, IL-6, IL-8). The results showed that the content of inflammatory factors decreased under the experimental conditions, and the collagen-chitosan mixed solution had the strongest inhibitory effect, indicating that the main components of the dressing had a certain effect on the control of the initial inflammatory response and prevention of further wound injury. An *in vivo* study showed that the contents of inflammatory factors (TNF-α, IL-1β) in the wound tissue of the control group were higher than those in the CCD and AgNP-CCD groups after scalding. These *in vivo* results were similar to those obtained *in vitro*, indicating that the prepared dressings had a confirmative effect on the control of the inflammatory response and a great benefit for wound healing.

Ma *et al*. [22] revealed that the collagen-chitosan scaffold could sufficiently support and accelerate fibroblast infiltration from the surrounding tissues in animal tests. Moreover, You *et al*. [38] confirmed the wound healing acceleration effects of a silver nanoparticle-loaded collagen/chitosan scaffold. We found that although there was no significant difference in the healing rate among these groups within the first 7 days after scalding, on Day 10, wound healing was significantly accelerated in the groups treated with CCD and AgNP-CCD. HE staining results also showed that on Day 7 after scalding, inflammatory cell infiltration and granulation tissue formation were observed on wounds that were treated with CCD or AgNP-CCD, as well as neovascularization and epithelialization. On Day 14, the wound tissue structure was clear with complete epithelialization from the above two dressings, indicating that CCD and AgNP-CCD could significantly promote deep second-degree burn wound healing.

Silver is a powerful antibacterial agent with efficient and wide-spectrum antibacterial functions. AgNPs can destroy membranes easily, pass into the microbial body and convert into silver ions (Ag^+^) in the cytoplasm, damaging the intracellular structure as a secondary result [39]. The results showed that the lower concentration of AgNP solution (2.5 mg/l) had a limited inhibitory effect on HUVECs and no effect on the secretion of growth and inflammatory factors, which is in accordance with previous reports [40]. The results also showed that CCD had certain antibacterial activity against *S. aureus*, *E. coli* and *P. aeruginosa*, and AgNP-CCD (with AgNPs >0.3 mg/cm^2^) exhibited an antibacterial rate >99% against these pathogens, which was similar to previous reports [41]. Potara *et al.* [42] showed a synergistic antibacterial activity resulting from the combination of chitosan and AgNPs; that is, the antibacterial effect of AgNPs and the electrostatic binding of chitosan. During the whole healing process, there was no significant difference between AgNP-CCD and CCD in the healing rate and the levels of growth and inflammatory factors, indicating that the addition of AgNPs did not affect the positive effects of the prepared dressing on wound healing.

## Conclusion

The proper proportion and concentration of the collagen-chitosan mixed solution were effective in promoting cell proliferation and regulating the levels of cytokines. The prepared silver nanoparticle-loaded CCDs demonstrated satisfactory morphological and physical properties as well as antibacterial activities, playing an important role in the promotion of deep second-degree burn wound healing and regulation of growth and inflammatory factors.
